# Olivocochlear efferent function: issues regarding methods and the interpretation of results

**DOI:** 10.3389/fnsys.2014.00142

**Published:** 2014-08-12

**Authors:** John J. Guinan Jr.

**Affiliations:** ^1^Eaton Peabody Laboratory of Auditory Physiology, Department of Otolaryngology, Massachusetts Eye and Ear InfirmaryBoston, MA, USA; ^2^Department of Otology and Laryngology, Harvard Medical SchoolBoston, Massachusetts, USA

**Keywords:** olivocochlear efferents, medial olivocochlear, MOC, attention, task difficulty, auditory psychophysics

## Abstract

As studies of the olivocochlear (OC) efferent system have matured, issues have been identified that need to be taken into account in the design of new studies and in the interpretation of existing work. The need for high signal-to-noise ratios (SNRs), multiple alternations of conditions, and avoiding middle-ear-muscle activation have been previously highlighted. Less well-known issues include: Contralateral medial OC (MOC) effects may not be good proxies for ipsilateral (ipsi) MOC effects; MOC-induced changes in otoacoustic emissions (OAEs) may not accurately show MOC-induced changes in auditory-nerve (AN) responses; measuring OAE differences from before to after psychophysical trials yields the transient OAE change but not tonic MOC activation; tonic MOC activation may be measurable by several techniques including by OAE differences in trials in which the subject’s judgment was correct vs. trials that were incorrect; SNRs can be preserved by Bootstrap statistical tests; differences in task difficulty may outweigh differences in subject attention; lateral efferent effects are little understood and may be tied to MOC effects; to assess whether MOC strength predicts protection from acoustic trauma, prospective tests in humans are needed.

## Introduction

The goal of this perspective is to provide little-appreciated observations that will help in interpreting the existing efferent literature and in guiding future work. To do this, some general review is necessary (for references see: Guinan, 1996, 2006, 2012a).

The olivocochlear (OC) efferents consist of medial (MOC) and lateral (LOC) groups that innervate outer hair cells (OHCs) and auditory-nerve (AN) dendrites under inner hair cells (IHCs), respectively. MOC neurons with crossed and uncrossed projections to the cochlea both receive inputs from the opposite cochlear nucleus and form MOC reflexes (MOCRs). The result is that uncrossed fibers mediate the contralateral (contra) MOCR, and crossed fibers mediate the ipsilateral (ipsi) MOCR (which is a double-crossed reflex) (Liberman and Brown, 1986).

## Do ipsilateral and contralateral sounds produce similar effects in the cochlea?

In most psychophysical studies of the detection of a signal in noise, the signal and noise are both in the same ear, the ipsi ear, so the noise elicits MOC activity through the ipsi MOCR. However, for technical reasons, most human physiologic studies monitor MOC effects using otoacoustic emissions (OAE) in the ipsi ear, but activate MOC efferents with contra noise. Using such a paradigm to correlate across-subject MOCR strengths with signal-in-noise discriminations is comparing contra MOCR strengths to ipsi MOCR effects.

Ipsi and contra MOCRs may be different in the brainstem and/or in the cochlea. Crossed and uncrossed MOC efferents originate in similar brainstem regions and appear to terminate on OHCs in similar ways. Anatomically, no difference in crossed and uncrossed MOC fibers has been described regarding where on OHCs they synapse or in the transmitters they use, but many anatomical experiments were not able to distinguish crossed from uncrossed MOC fibers. One ipsi/contra difference is that relative to ipsi MOCR fibers, contra MOCR fibers have an apical offset in their OHC terminations and a larger span of innervation (Brown, 2014). MOC fibers also terminate on type II auditory nerve fibers within the organ of Corti, and, in some species, on spiral-ganglion cells (Rask-Andersen et al., 2000; Thiers et al., 2008). Both of these are places where ipsi and contra reflex innervation may be different.

Physiological evidence shows ipsi-contra similarities and differences in MOC activation and MOC effects. Most experiments have been only done on one species, but anatomical results suggest there may be little difference in these results across most mammalian species. In cats, the MOC inhibition of AN responses is similar in magnitude for crossed and uncrossed MOC fibers on an inhibition-per-MOC-fiber basis (Gifford and Guinan, 1987). In humans, OAE changes are twice as large for ipsi noise as for contra noise when the MOC-elicitors are half-octave-bands—which is consistent with a 2 to 1 ratio of ipsi to contra MOCR fibers. However, ipsi and contra noises elicit similar-amplitude OAE inhibitions when the noises are broad band (Lilaonitkul and Guinan, 2009). Thus the growth of MOCR activation with bandwidth is different for ipsi and contra MOCRs, which means that in the brainstem, the reflexes’ neural summation across frequency is different. Within the cochlea, ipsi and contra MOCRs produce different relationships between the changes in OAE phase and OAE amplitude (Lilaonitkul and Guinan, 2012). Overall, while ipsi and contra MOCRs show many similarities, in summation across frequency, cochlear effects, and cochlear innervation anatomy, they are different.

Does measuring contra-MOCR strength adequately substitute for measuring ipsi-MOCR strength? Contra-MOCR strength varies greatly across humans (Backus and Guinan, 2007). It seems reasonable to expect ipsi and contra reflex strengths to vary similarly across subjects and to be correlated. However, since the reflexes are different in some respects, it cannot be assumed that their strengths are strongly correlated.

## MOC-induced changes in OAEs vs. in Auditory Nerve (AN) responses

MOC activation in humans can be assessed by the MOC-induced changes in OAEs, but how well changes in OAEs represent changes in neural responses is not known. The important MOC-induced functional change is in the neural response. MOC-induced OAE and neural changes have been compared in a few studies that suggest that OAE changes are not good indicators of neural changes (Puria et al., 1996; Chabert et al., 2002; Zhao et al., 2012; Lichtenhan et al., 2014).

The changes with sound level of MOC effects on OAEs vs. on neural responses are informative. Many studies have shown that MOC-induced changes in basilar membrane (BM) responses, OAEs, and AN compound potentials (CAPs) are largest at threshold and decrease as sound level is increased. For AN fibers with high spontaneous rates (SRs), MOC-induced inhibition is greatest at low sound levels, but for low-SR fibers, MOC inhibition is highest at mid-to-high sound levels (Guinan and Stankovic, 1996). It might seem that MOC effects on low-SR fibers can be ignored because they are only a small fraction of AN fibers. However, low-SR fibers are the main ones whose firing rate grows at mid-to-high sound levels, and their responses most likely play an outsized role in behavioral discriminations at these levels. Additionally, the motion at the top of the organ of Corti is different from BM motion in tuning and growth with sound level (Zha et al., 2012). Motion at the top of the organ of Corti drives IHCs, not BM motion. Finally, the coupling between organ of Corti motion and the drive to IHC stereocilia is complex (Guinan, 2012b). Overall, one should beware of thinking that the MOC effects on AN responses (and thus, in psychophysical tests) follow MOC inhibition of BM motion and are always largest at low sound levels.

## Issues in comparing MOC effects and psychophysical performance

As noted earlier, contra MOCR measurements may not accurately represent ipsi MOCR activation, so comparisons of psychophysical performance and MOC activation are best done using the ipsi MOCR. How is the ipsi MOCR to be measured? One possible test is the DPOAE-onset-adaptation test (Liberman et al., 1996). In humans (where, in contrast to animals, efferents cannot be cut as a control), this test does not distinguish MOC effects from intrinsic cochlear adaptation. The heavy dominance of ipsi adaptation over contra adaptation in humans (Kim et al., 2001) indicates that human ipsi DPOAE adaptation contains a large intrinsic component. Another technique that works in animals but not humans is measurement of DPOAE 2F1-F2 adaptation (F1 and F2 are the primary-tone frequencies) at frequencies near DPOAE response dips. In animals dips arise from OHC stereocilia nonlinearity (Lukashkin and Russell, 2002) and the MOCR directly changes OHC properties. In contrast, in humans most dips are due to interference between DPOAE distortion and reflection components (Talmadge et al., 1999) and the depth of these dips is due to how well they cancel which is only indirectly affected by the MOCR.

Another issue in measuring ipsi MOCR effects is that ipsi elicitor sounds suppress concurrent ipsi OAEs. This can be avoided by using MOCR elicitors with no energy near the OAE-probe frequency, but such a choice may not be compatible with the psychophysical test. For most psychophysical tests (e.g., signal-in-noise discriminations), ipsi OAE measurements cannot be done simultaneously with the stimuli being discriminated. An alternative is to measure MOC effects from OAEs before and immediately after each psychophysical trial. The difference in OAE amplitudes from before to after the trial provides a measure of the trial-locked *change* in MOC activation. However, the difference misses any tonic MOC activation, i.e., MOC activation that begins at the beginning of a block of trials and continues throughout the block during both the pre- and post-trial measurements.

If the subject’s mental getting ready at the beginning of a task brings about tonic MOC activation (presumably from cortical activation of descending pathways), then when the same sounds are heard without a task (called “passive listening”), perhaps there is no tonic MOC activation. In passive listening, the difference between OAE measurements before and after the trial sounds is from transient MOC activation. If during pre-measurements in passive listening there is no tonic activation, then any OAE differences in the pre-trial measurements from active compared to passive listening would reveal tonic MOC activation during the active-listening trials. To avoid effects of drift and differences across measurement sessions, interleaving blocks of task and no-task trials within a measurement session is probably necessary. On the interleaved no-task blocks, the subject must “relax” and not attempt discriminations, i.e., not tonically activate their efferents. A long training period may be required for subjects to achieve good performance in tasks with alternation of MOC activation. Not all subjects may be able to do this, i.e., to achieve performance on blocks alternating MOC-on/MOC-off that equals their performance when doing long sequences of just MOC-on or just MOC-off.

One method to reveal tonic MOC activation would be to interleave a task that has a MOC perceptual benefit and a task in which MOC activation would be detrimental (e.g., detecting a tone in quiet at a frequency far from spontaneous OAEs (SOAEs)—see Dewey et al., 2014). Optimum performance would require MOC activation in the first case, and turning-off MOC activation in the second case. Alternating these would allow tonic MOC activation to be detected by comparing the pre-trial OAE levels. Again, a long training period may be required to achieve good performances. Whenever there is long training, presumably there is learning involved and this may change the OAE results over time and make it difficult to obtain the stationary periods necessary for good averaging.

A powerful but difficult to apply method is the correct/incorrect comparison. In a series of trials using identical stimuli, some subject judgments are correct and some are incorrect. Since the stimuli are the same (with random permutations in presentation order), differences in subject judgments are presumably due to internal variations within the subject (e.g., in subject alertness, MOC activation, etc.). If correct trials, on average, have more MOC activation than incorrect trials, this would be strong evidence that the MOC activation actually produced the perceptual benefit, since all stimulus variables are the same (although correlation doesn’t prove causation). With this method, transient activation during each trial is shown by the difference in before-trial to after-trial OAE amplitudes, and variation in tonic activation may be revealed by comparing the pre-trial OAE amplitudes from correct vs. incorrect trials. A variant of this method was used in chinchillas who skipped making a choice on many trials (which is not allowed in the normal human paradigm) with the result that a difference in MOC activation was found between trials when the animal made a choice vs. the skipped trials, but not between correct and incorrect trials (Delano et al., 2007).

## Statistical tests

Both the pre-to-post trial method and the correct/incorrect comparison method require many trials to achieve adequate signal-to-noise ratios (SNRs). The best SNR is achieved by computing differences using *all* of the available trials. If the data are broken into N subsets to have N tokens for a statistical test, then each token has poorer SNR than the grand average. One way to keep the highest SNR is to use a bootstrap method.

The bootstrap method is described initially with a correct/incorrect comparison. First, OAEs should have passed SNR criteria to remove those with excessive noise. Also, to minimize effects of drift, pre and post data from a trial should both be used or both excluded. Suppose there are Nc and Ni correct and incorrect noise-minimized trials: (1) Pre-trial and post-trial OAE averages are done using all of the Nc trials and separately using all of the Ni trials. The statistic of interest (the “real-stat”) is then computed from these averages. (2) The null-hypothesis is that there is no difference between Nc and Ni trials, so all trials are pooled (i.e., their actual correct/incorrect value is ignored). From this pool, Nc trials are randomly chosen to form a pseudo-Nc set which are averaged. The remaining Ni trials become the pseudo-Ni set and are averaged. Both selections must be done without replacement so the noise from a trial is never added in twice (different random noises add orthogonally, but the same noise added to itself adds linearly and would produce a noise summation that is not equivalent to the noise summation in step 1). The statistic of interest (the “pseudo-stat”) is then computed from these averages exactly as in step 1. (3) Step 2 is done 1000 times (10,000 is even better), each with a different randomization, yielding 1000 pseudo-stats which show the “null-hypothesis” distribution. (4) If there are fewer than 50 out of 1000 pseudo-stats that have more extreme values than the real-stat, then the real stat is statistically significant at the 0.05 level. Since usually Nc > Ni, the average of the correct trials will generally have a lower noise level than the average of the incorrect trials. However, no noise correction is needed in the statistical test because the real and pseudo averages use the same number of trials. However, the Nc > Ni difference in noise level can bias the average difference and needs to be considered in the interpretation of this difference. Control computations should be done to check that the Nc and Ni noise-levels-per-trial are not different and to inform the interpretation. If instead of correct/incorrect, the pre/post difference is tested, then the null hypothesis is that there is no pre/post difference. On each trial, pre and post values are pooled and are randomly assigned to be pseudo-pre and pseudo-post when calculating the pseudo distributions. The rest of the bootstrap is done as in the correct/incorrect comparison.

## Task difficulty and the comparison of task/no-task conditions

A potentially important but little considered issue is task difficulty. It is well established that the pupillary reflex varies with task difficulty, including for auditory tasks (Kahneman and Beatty, 1966; Zekveld and Kramer, 2014). Like the pupillary reflex, the MOCR is a brainstem-level reflex that receives descending inputs. Furthermore, MOC activation has been shown to vary with task difficulty (Delano et al., 2007). To validate that a difference in MOC activation is due to selective attention and not to task difficulty, the task difficulty must be kept constant in the tasks compared. This might be done by setting the parameters so the compared tasks yield the same percentage of correct trials. Reports that claim to show differences in MOC activation due to selective attention (e.g., differences ascribed to auditory vs. visual attention) should be examined closely to determine if the supposed selective attention difference may actually be from differences in task difficulty.

It is well known that pushing a button can cause noise. Less well known is that subjects sometimes make small settling movements at the beginning of trials that add noise at the earphone. Large noises from gross motion can be removed easily by an artifact rejection system. However, small noises (which can be revealed by averaging two adjacent responses after reversing one) vary in amplitude over a wide range and are difficult to reject. Care must be taken to insure that differences between pre and post-task OAEs are not contaminated by differences in pre-post noise levels.

There are several areas of the MOC literature in which the existing reports appear to give contradictory results (e.g., attention increases MOC activation, or decreases MOC activation). Some of these may become clearer when the above considerations are taken into account. Less weight should be given to studies that have methodological deficiencies based on the issues presented above. However, even well-done studies can be expected to depend on the stimulus parameters explored and reported differences may be due to difference in the parameters used. Too many studies look only at one condition, or a very few conditions, and then state conclusions as if these conclusions apply widely. More studies are needed that vary the sound parameters over a large enough range to capture how the MOC effect varies with the parameters.

## LOC function and MOC-LOC interactions

There is good evidence that LOC activity reduces acoustic trauma and auditory aging (Liberman et al., 2014). There is no direct evidence for activation of LOC neurons by sound, but it is presumed they respond to sound, in part because they are located in a brainstem auditory nucleus. Neurons in this brainstem region are excited by ipsi sound and inhibited by contra sound. If LOC neurons respond with the same laterality pattern, then those that inhibit AN fibers (some LOCs excite and some inhibit), may balance left-right cochlear outputs to optimize sound localization from interaural intensity differences (Guinan, 1996). Darrow et al. (2006) presented data favoring this hypothesis, but Larsen and Liberman (2010) presented data opposing this hypothesis. LSO fibers are unmyelinated, conduct slowly and change AN firing over the course of minutes (Groff and Liberman, 2003) so whatever they do is likely to be on a slow time scale.

There are several ways in which the MOC and LOC systems interact. MOC activity reduces cochlear amplifier gain which reduces AN firing rates, and thus MOC activity may reduce LOC activity. Likewise, LOC fibers change AN activity and thus influence MOC firing. MOC-LOC interaction also occurs within the tunnel of Corti where LOC fibers synapse on MOC fibers (Liberman, 1980), but it is unknown whether these synapses are excitatory or inhibitory. Overall, there is ample opportunity for MOC-LOC interactions so that their effects may be correlated across subjects and circumstances. Thus, effects attributed to one system, may be due in part to, or influenced by, the other system.

## Tests to predict susceptibility to acoustic trauma and auditory aging: issues

Considering the strong evidence that the MOC and LOC systems help protect from both acoustic trauma and auditory aging, it would be highly desirable to have tests for MOC and LOC function. At present, there is no LOC test, but MOC function can be tested in humans by sound-evoked MOC effects on OAEs. Many prior studies of MOC function in humans used group averages, but for prediction in an individual the MOC test must be accurate in the individual. This requires achieving an adequate SNR in each subject, something which is especially difficult when small MOC effects are to be measured in the most-vulnerable subjects. SNRs of 25 dB or more are likely to be needed (e.g., Goodman et al., 2013, Figure 8). To minimize test time, an averaging stopping rule should be used that is based on achieving the SNR to detect a pre-determined low-amplitude MOC effect (Guinan, 2006, 2012a). One little-discussed issue is: “How many frequencies need to be tested?” Related questions are: “How much does MOC activation vary across frequency in different subjects?” What does finding MOC activation at an easy-to-test frequency (e.g., one with large OAEs) indicate about the MOC activation at hard-to-test frequencies? More work on these questions is needed.

One possibility is that MOC activation at non-traumatic levels is aimed at gaining perceptual benefits, but for protection at very high sound levels other brainstem mechanisms are activated, similar to those that activate the middle-ear-muscle system. The relative strengths of such low-level and high-level MOC systems may vary across individuals. Animal work indicates that MOC tests at low sound levels can be predictive of MOC anti-trauma strength (Maison and Liberman, 2000) but this does not rule out low-to-high-level differences. Prospective tests in humans are needed to show how well MOC tests predict susceptibility to acoustic trauma, e.g., MOC tests applied at the start of a person’s work in a loud-sound environment (e.g., Lapsley Miller et al., 2006).

## Conclusion

There are many potential problems in studying efferents. Some have clear solutions. Others do not. In all cases it is important to be aware of the potential problems so that experiments can be designed well and interpreted properly.

## Conflict of interest statement

The author declares that the research was conducted in the absence of any commercial or financial relationships that could be construed as a potential conflict of interest.
